# Tooth Loss and Nutritional Status in 120,994 Children Aged 6–9 Years in Mongolia: A Population-Based Study

**DOI:** 10.3390/children13020191

**Published:** 2026-01-29

**Authors:** Batbold Gan-Ochir, Oyuntugs Byambasukh, Batzorig Bayartsogt, Enkh-Orchlon Batbayar, Enkhtur Yadamsuren, Damdindorj Boldbaatar, Khurelbaatar Nyamdavaa, Ganbayar Luuzanbadam, Otgonbaatar Jugder, Delgertsetseg Jargaltsogt, Oyunsuren Enebish

**Affiliations:** 1 Department of Oral and Maxillofacial Surgery, School of Dentistry, Mongolian National University of Medical Sciences, Ulaanbaatar 14210, Mongolia; batbold@mnums.edu.mn (B.G.-O.); enkhorchlon@mnums.edu.mn (E.-O.B.); 2 Department of Endocrinology, School of Medicine, Mongolian National University of Medical Sciences, Ulaanbaatar 14210, Mongolia; oyuntugs@mnums.edu.mn; 3 Department of Epidemiology and Biostatistics, School of Public Health, Mongolian National University of Medical Sciences, Ulaanbaatar 14210, Mongolia; batzorig@mnums.edu.mn; 4 Department of Administration, Mongolian National University of Medical Sciences, Ulaanbaatar 14210, Mongolia; enkhtur@mnums.edu.mn (E.Y.); damdindorj@mnums.edu.mn (D.B.); khurelbaatar.n@mnums.edu.mn (K.N.); 5 National Center for Maternal and Child Health of Mongolia, Ulaanbaatar 16060, Mongolia; ganbayar.l@ncmch.gov.mn (G.L.); otgonbaatar.j@ncmch.gov.mn (O.J.); 6 Department of Dental Hygiene, School of Dentistry, Mongolian National University of Medical Sciences, Ulaanbaatar 14210, Mongolia; 7 Ministry of Health, Ulaanbaatar 14253, Mongolia

**Keywords:** tooth loss, nutritional status, oral health, weight-for-height Z-score

## Abstract

**Highlights:**

**What are the main findings?**

- Tooth loss was independently associated with lower weight-for-height and weight-for-age Z-scores among children aged 6–9 years.
- A dose-response pattern was observed, with progressively lower nutritional indicators as the number of extracted teeth increased.

**What are the implications of the main findings?**

- Tooth loss in early childhood may serve as a marker of vulnerability to suboptimal nutritional status.
- Integrated oral health and nutrition strategies may help mitigate growth-related risks in school-aged children.

**Abstract:**

**Background:** Tooth loss in childhood reflects cumulative oral disease and may impair dietary intake, potentially influencing nutritional status. Evidence on the association between tooth loss and anthropometric indicators in school-aged children remains limited. **Methods:** This population-based cross-sectional study used data from a nationwide health screening conducted by the Ministry of Health of Mongolia in 2023–2024. A total of 120,994 children aged 6–9 years were included. Tooth loss was categorized as 0, 1–2, or ≥3 extracted teeth. Nutritional status was assessed using weight-for-height (WHZ), weight-for-age (WAZ), height-for-age (HAZ) Z-scores, and body mass index (BMI). Associations between tooth loss and anthropometric indicators were examined using unadjusted and multivariable linear regression models adjusting for age and sex. **Results:** Overall, 12.5% of children had experienced tooth loss. Mean WHZ and WAZ decreased progressively with increasing tooth loss. In adjusted analyses, children with 1–2 extracted teeth had lower WHZ (β = −0.025; 95% CI: −0.047 to −0.004), and those with ≥3 extracted teeth had substantially lower WHZ (β = −0.058; 95% CI: −0.084 to −0.032), compared with children without tooth loss. Similar associations were observed for WAZ. No significant associations were found between tooth loss and BMI or HAZ. No interactions with age or sex were detected. **Conclusions:** Tooth loss was independently associated with lower indicators of current nutritional status among children aged 6–9 years. These findings underscore the importance of integrating oral health and nutrition strategies in childhood health programs.

## 1. Introduction

Oral health is an integral component of child health and development worldwide [1]. Dental caries remains one of the most prevalent chronic conditions in children and is a leading cause of pain, infection, and tooth loss even at young ages [2,3]. Globally, untreated dental caries in primary teeth affects hundreds of millions of children and represents a substantial public health burden [4]. Beyond oral symptoms, poor oral health may impair mastication, influence food choices, and reduce dietary intake, potentially affecting nutritional status during critical periods of growth and development [5,6,7].

Mongolia bears one of the highest reported burdens of childhood dental caries globally. National and regional studies consistently demonstrate that more than three-quarters of young children are affected at an early age. For example, surveys among children under five years have reported caries prevalence ranging from approximately 76% to over 89%, even among predominantly middle socioeconomic groups, indicating widespread disease across population strata [8]. National oral health survey data further show that approximately 83% of five-year-old children in Mongolia have experienced dental caries, with a mean dmft exceeding five teeth, substantially higher than levels reported in many other countries in the Western Pacific and Central Asian regions [9]. In contrast, global estimates suggest that the prevalence of caries in primary teeth is generally lower, with marked heterogeneity across regions, but substantially fewer children experience severe disease in early childhood.

In addition to the overall disease burden, oral health inequalities are pronounced in Mongolia. Socioeconomic disparities in caries experience have been documented among children living in Ulaanbaatar, with lower household income strongly associated with higher caries prevalence and severity [10]. Similar social gradients in oral health have been observed globally, with children from disadvantaged backgrounds consistently experiencing worse oral health outcomes than their more advantaged peers [11,12]. These inequalities suggest that oral disease in children is socially patterned and may cluster among vulnerable populations, potentially contributing to broader health consequences, including compromised nutrition.

Early childhood caries is highly prevalent in Mongolia and represents an important precursor to later oral health outcomes. Studies among children under five years of age have shown persistently high caries prevalence despite national oral health programs, with behavioral and parental factors playing a significant role in disease development [13]. Severe caries in early life may lead to premature tooth extraction, resulting in tooth loss during early and middle childhood [1,2,3]. Tooth loss reflects cumulative oral disease and may represent a more severe and functionally relevant marker of oral health burden than caries alone.

Adequate nutrition is essential for optimal physical growth and development in childhood. Anthropometric indicators such as weight-for-height, weight-for-age, and height-for-age Z-scores are widely used to assess children’s nutritional status, reflecting acute, composite, and chronic growth patterns, respectively [14,15,16]. Factors that interfere with normal eating behaviors or food choices may therefore influence these indicators. Poor oral health, including tooth loss, may reduce masticatory efficiency and contribute to altered dietary intake, particularly in younger children who are still developing stable eating habits [6,7,17].

Previous studies have reported associations between dental caries and undernutrition in children, especially in low- and middle-income countries [18,19]. However, existing evidence has several limitations. Many studies focus on the presence of caries rather than tooth loss itself, despite tooth loss representing a more advanced and cumulative outcome of oral disease. In addition, nutritional status is often assessed using categorical outcomes such as underweight or obesity, which may obscure more subtle associations detectable through continuous anthropometric measures [20,21]. Consequently, evidence on the relationship between tooth loss and nutritional status in school-aged children remains limited and inconsistent.

Large, population-based health screening programs provide an opportunity to examine this relationship more comprehensively. Countries undergoing rapid social and dietary transitions offer particularly informative settings, as children may be simultaneously exposed to persistent oral health challenges and changing nutritional environments [22]. Mongolia has experienced substantial social and dietary changes in recent decades, with implications for both oral health and child nutrition.

In this context, the present study used data from a nationwide health screening program to examine the association between tooth loss and anthropometric indicators among children aged 6–9 years in Mongolia. Using a large population-based sample, we aimed to assess whether increasing levels of tooth loss were associated with lower indicators of nutritional status, with particular emphasis on weight-related Z-scores.

## 2. Materials and Methods

### 2.1. Study Design and Study Population

This study was a population-based cross-sectional analysis using data from a nationwide child health screening program conducted by the Ministry of Health of Mongolia between 2023 and 2024. The screening was implemented across all regions of the country as part of routine public health surveillance and aimed to assess the health, nutritional status, and oral health of school-aged children using standardized procedures. The present study represents a secondary analysis of anonymized data derived from this national screening program.

Children aged 6–9 years who participated in the national health screening during the study period were eligible for inclusion. Records were included if information was available on age, sex, anthropometric measurements, and oral health indicators. Exclusion criteria were missing or incomplete data on key variables, including age, sex, height, weight, or tooth loss; biologically implausible anthropometric values or Z-scores identified using World Health Organization (WHO) recommended cut-offs; and extreme outliers likely to reflect measurement or data entry errors.

The national health screening program was conducted by the Ministry of Health of Mongolia [23] as part of routine public health activities (Approval No: 23/042, dated 5 July 2023). The dataset used for this study was anonymized prior to analysis. The secondary analysis of de-identified data was conducted in accordance with national regulations and ethical standards.

### 2.2. Oral Health Assessment

Oral health examinations were conducted as part of the national health screening program by trained dentists or dental health professionals using standardized examination protocols issued by the Ministry of Health of Mongolia. Examinations were performed under field conditions using basic dental instruments and adequate lighting, in accordance with routine public health practice.

Tooth loss was assessed as the number of teeth that had been extracted prior to the examination and were absent at the time of assessment. Extractions were recorded irrespective of the underlying reason; however, in this age group, tooth loss primarily reflects extractions due to dental caries. The number of extracted teeth was recorded as a discrete count variable.

For analytical purposes, tooth loss was categorized into three clinically interpretable groups:

- 0 extracted teeth, indicating no history of tooth loss;
- 1–2 extracted teeth, indicating limited tooth loss;
- ≥3 extracted teeth, indicating more extensive tooth loss.

Additional oral health indicators were recorded, including the number of decayed teeth and the number of filled teeth. The decayed-missing-filled teeth (DMFT) index was calculated as the sum of decayed, missing, and filled teeth, providing a composite measure of cumulative caries experience.

### 2.3. Anthropometric Measurements and Nutritional Indicators

Anthropometric measurements were obtained by trained healthcare personnel following standardized measurement procedures. Body weight was measured to the nearest 0.1 kg using calibrated digital scales, with children wearing light clothing and no shoes. Standing height was measured to the nearest 0.1 cm using portable stadiometers, with children standing upright and barefoot.

Body mass index (BMI) was calculated as weight in kilograms divided by height in meters squared (kg/m^2^).

Nutritional status was assessed using the World Health Organization (WHO) child growth standards. Age- and sex-specific Z-scores were calculated for [15]:

- Weight-for-height Z-score (WHZ), reflecting current or acute nutritional status;
- Weight-for-age Z-score (WAZ), reflecting a composite measure of weight relative to age;
- Height-for-age Z-score (HAZ), reflecting chronic growth status and long-term nutritional adequacy.

Z-scores were calculated using WHO reference values and treated as continuous variables in all analyses to preserve statistical power and allow detection of subtle differences in growth patterns. Biologically implausible Z-scores were identified using WHO-recommended cut-offs and excluded from the analysis as part of data cleaning procedures.

### 2.4. Covariates

Covariates included age (years) and sex, which were selected a priori based on their established associations with both oral health and nutritional status. Residence (urban/rural) was considered in descriptive analyses. Interaction terms between tooth loss and age and sex were evaluated to assess potential effect modification.

### 2.5. Statistical Analysis

Descriptive statistics were used to summarize participant characteristics. Continuous variables were presented as means with standard deviations or medians with interquartile ranges, as appropriate. Differences in anthropometric and oral health indicators by age group and sex were assessed using one-way analysis of variance (ANOVA).

Associations between tooth loss and anthropometric indicators were examined using linear regression models. The tooth loss category was entered as a set of dummy variables, with children without extracted teeth serving as the reference group. Both unadjusted models and models adjusted for age and sex were fitted. Regression results were reported as unstandardized coefficients (β) with 95% confidence intervals (CI).

Adjusted estimated marginal means of WHZ by tooth loss category were derived from analysis of covariance models. Interaction terms between tooth loss and age and sex were tested.

All statistical analyses were performed using IBM SPSS version 28.0 (IBM Corp., Armonk, NY, USA). A *p*-value of <0.05 was considered statistically significant for all analyses.

## 3. Results

A total of 120,994 children aged 6–9 years were included in the analysis. The mean age was 7.52 ± 1.11 years, and 50.2% were boys. Most children resided in urban areas (63.5%). Overall, 12.5% of children had experienced tooth loss, defined as the presence of at least one extracted tooth. The majority of children (87.5%) had no extracted teeth. Among those with tooth loss, 10.5% had one to two missing teeth, while 2.0% had three or more missing teeth.

Anthropometric indicators were generally close to World Health Organization reference values. The mean HAZ was 0.01 ± 1.00, the mean WAZ was 0.00 ± 1.00, and the mean WHZ was −0.04 ± 1.00. Mean BMI was 16.73 ± 2.30 kg/m^2^ (Table 1). Distributions of anthropometric Z-scores were approximately symmetric, with medians close to zero and interquartile ranges of 1.31 for HAZ, 1.25 for WAZ, and 1.21 for WHZ. BMI showed moderate right skewness, with a median of 16.38 kg/m^2^ and an interquartile range of 2.69 kg/m^2^.

Oral health indicators demonstrated substantial variability. The mean number of decayed teeth was 2.22 ± 2.59, with a median of 2 teeth (IQR: 3). The number of filled teeth was low, with a mean of 0.63 ± 1.30 and a median of 0 (IQR: 1). The mean number of extracted teeth was 0.22 ± 0.70, with a median of 0, indicating that most children had not experienced tooth loss. The mean DMFT index was 3.07 ± 3.03, with a median of 2 (IQR: 5). Dental caries was common in the study population, with 73.49% of children presenting with at least one decayed tooth.

Anthropometric and oral health indicators varied across the 6–9-year age range (Table 2). Mean WHZ increased with age, with lower values observed at age 6 and progressively higher values at ages 7–9 (*p* < 0.001). A similar age-related pattern was observed for BMI, which increased steadily from age 6 to age 9 (*p* < 0.001). In contrast, mean HAZ and WAZ values remained stable across age groups, with no statistically significant differences observed (HAZ: *p* = 0.551; WAZ: *p* = 0.998). Clear age-related differences were observed for oral health indicators. The mean number of decayed teeth decreased with increasing age, while the mean number of filled teeth showed modest variation across age groups. The mean number of extracted teeth increased slightly with age, although overall levels of tooth loss remained low across all ages. The DMFT index also declined with age, reflecting changes in the distribution of decayed, missing, and filled teeth over time (all *p* < 0.001).

Anthropometric indicators differed modestly by sex. Mean WHZ was slightly lower among boys than girls, while BMI was higher among boys. No meaningful differences were observed between boys and girls for HAZ or WAZ. Oral health indicators showed clearer sex differences. Boys had higher mean numbers of decayed teeth and higher DMFT index values compared with girls, whereas girls had slightly higher numbers of filled teeth. The number of extracted teeth also differed by sex, although overall values remained low in both groups.

Mean anthropometric indicators differed modestly across tooth loss categories (Table 3). Mean WHZ decreased progressively with increasing tooth loss, from −0.03 ± 1.00 among children without tooth loss to −0.06 ± 0.99 among those with one to two missing teeth and −0.09 ± 0.94 among those with three or more missing teeth (*p* = 0.001). A similar but smaller pattern was observed for WAZ (*p* = 0.004). In contrast, mean BMI and HAZ did not differ significantly across tooth loss categories.

**Figure 1:**
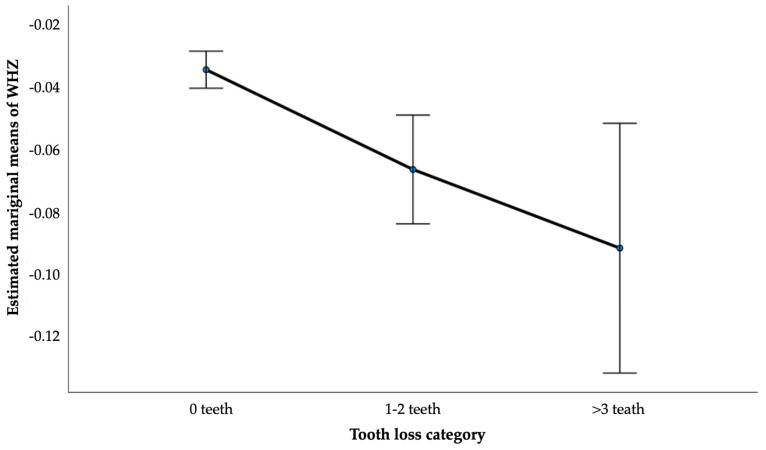
Adjusted estimated marginal means of WHZ by tooth loss category among children aged 6–9 years.

Compared with children without tooth loss, those with one to two extracted teeth had a 0.025 SD lower WHZ, while children with three or more extracted teeth had a 0.058 SD lower WHZ, indicating a dose–response relationship. Similar associations were observed for WAZ, whereas no significant associations were found for BMI or HAZ. No significant interactions between tooth loss and age or sex were detected. Adjusted estimated marginal means further illustrated a stepwise decrease in WHZ with increasing tooth loss (Figure 1).

In unadjusted analyses, children with tooth loss had lower anthropometric Z-scores compared with those without tooth loss (Table 4). For weight-for-height Z-score (WHZ), both 1–2 extracted teeth (β = −0.026; 95% CI: −0.045 to −0.008) and ≥3 extracted teeth (β = −0.052; 95% CI: −0.091 to −0.012) were associated with lower WHZ. These associations remained statistically significant after adjustment for age and sex, with evidence of a dose–response relationship, particularly for children with ≥3 extracted teeth (adjusted β = −0.058; 95% CI: −0.084 to −0.032). Similarly, for the weight-for-age Z-score (WAZ), both categories of tooth loss were associated with lower scores compared with no tooth loss in unadjusted models. These associations were unchanged after adjustment, with adjusted β values of −0.026 (95% CI: −0.045 to −0.008) for 1–2 extracted teeth and −0.040 (95% CI: −0.080 to −0.000) for ≥3 extracted teeth. Overall, tooth loss was consistently associated with lower indicators of current nutritional status, while adjustment for age and sex did not materially alter the observed associations.

## 4. Discussion

In this large population-based study of children aged 6–9 years, tooth loss was independently associated with lower indicators of current nutritional status. Specifically, increasing levels of tooth loss were associated with progressively lower weight-for-height (WHZ) and weight-for-age (WAZ) Z-scores, even after adjustment for age and sex. A clear dose–response pattern was observed, with children experiencing greater tooth loss showing more pronounced reductions in these weight-related growth indicators. In contrast, no significant associations were found between tooth loss and height-for-age (HAZ) or body mass index (BMI).

The selective association with WHZ and WAZ is biologically plausible and consistent with existing conceptual frameworks linking oral health and nutrition [1,5]. WHZ and WAZ are sensitive to recent changes in dietary intake and energy balance, whereas HAZ reflects long-term linear growth and cumulative nutritional exposures [24]. Tooth loss in early childhood may compromise masticatory function and reduce chewing efficiency, leading children to avoid harder-to-chew but nutritionally important foods such as fruits, vegetables, and protein-rich items [6,25]. These dietary adaptations may result in subtle but measurable effects on weight-related growth without immediately affecting stature.

The absence of an association with BMI suggests that tooth loss may not be linked to extremes of underweight or overweight in this age group but rather to modest shifts in overall nutritional status. BMI is a composite indicator influenced by both height and weight and may be less sensitive to short-term changes in dietary intake than Z-score-based measures [26]. Similarly, the lack of association with HAZ is expected, as deficits in linear growth typically reflect chronic nutritional deprivation or long-standing health conditions rather than relatively recent oral health events [16]. Together, these findings highlight the importance of selecting appropriate anthropometric indicators when examining oral health-nutrition relationships in children.

Our findings are broadly consistent with previous studies reporting associations between poor oral health and undernutrition in children, although much of the existing literature has focused on dental caries rather than tooth loss specifically [27,28,29]. Tooth loss represents a more severe and cumulative consequence of oral disease and may therefore have greater functional implications than untreated caries alone. However, evidence on the relationship between tooth loss and nutritional status in school-aged children remains limited and inconsistent across settings [30]. By using a large, nationally representative dataset and examining tooth loss as a categorical exposure with clinically interpretable thresholds, the present study adds robust evidence to this underexplored area of child health research.

The large sample size and national coverage of the data are key strengths of this study. The use of standardized anthropometric measurements and WHO growth standards enhances comparability with other studies and supports the internal validity of the findings [14]. The consistency of associations across unadjusted and adjusted models, as well as the absence of significant interactions with age or sex, suggests that the observed relationship between tooth loss and nutritional status is stable across subgroups. Although the effect sizes were modest at the individual level, even small shifts in anthropometric Z-scores may have meaningful implications at the population level, particularly in contexts where dental caries and tooth loss are prevalent.

From a public health perspective, these findings underscore the interconnectedness of oral health and nutrition in childhood. Tooth loss may serve as a marker of cumulative oral disease and broader vulnerability, reflecting underlying social, behavioral, and environmental factors that also influence nutritional status [31]. Integrating oral health promotion with child nutrition and general health programs may therefore offer opportunities for synergistic benefits. Preventive strategies aimed at reducing dental caries, promoting timely dental care, and avoiding unnecessary tooth extraction could contribute not only to improved oral health outcomes but also to better growth and development in children. Although the observed differences in weight-related Z-scores associated with tooth loss were modest at the individual level, their public health relevance should be considered in the context of the population scale. Even small downward shifts in mean anthropometric indicators, when affecting large numbers of children, may translate into a meaningful increase in the proportion at risk of undernutrition or growth faltering. In settings such as Mongolia, where childhood oral disease is highly prevalent and cumulative, these subtle associations may contribute to broader population-level nutritional vulnerability. From a public health perspective, preventing early tooth loss could therefore have benefits that extend beyond oral health, reinforcing the value of integrated preventive strategies targeting both oral disease and child nutrition.

Several limitations should be considered when interpreting these results. First, the cross-sectional design precludes causal inference, and reverse causality cannot be excluded; children with poorer nutritional status may also be more susceptible to oral disease and subsequent tooth loss. Second, residual confounding by unmeasured factors such as socioeconomic status, dietary intake, oral hygiene practices, or access to dental care is possible. Although the models were adjusted for age and sex, the national screening dataset did not include detailed socioeconomic indicators (e.g., household income or parental education) or dietary intake variables. Given the well-established social patterning of both oral health and child nutrition, residual confounding by unmeasured socioeconomic and dietary factors cannot be excluded. Future studies incorporating comprehensive socioeconomic and nutritional data would allow for a more nuanced assessment of the pathways linking tooth loss and growth outcomes. Third, information on the reasons for tooth extraction was not available, limiting the ability to distinguish between caries-related and other causes of tooth loss. Finally, although the dataset was large and population-based, the measurement error inherent in routine screening programs cannot be completely excluded.

Future research should aim to clarify the temporal and causal relationships between tooth loss and nutritional status in children. Longitudinal studies are needed to determine whether tooth loss precedes changes in growth indicators or whether underlying nutritional vulnerabilities contribute to poorer oral health outcomes. Studies incorporating detailed dietary assessments, socioeconomic indicators, and oral health behaviors would help elucidate the pathways linking oral health and nutrition. In addition, evaluating the impact of oral health interventions on subsequent nutritional outcomes could inform evidence-based policies and integrated child health strategies.

## 5. Conclusions

In conclusion, this large population-based study demonstrates that tooth loss is independently associated with lower indicators of current nutritional status among children aged 6–9 years. The presence of a dose–response relationship underscores the potential relevance of tooth loss as a marker of vulnerability affecting child growth. These findings highlight the importance of early prevention of oral disease and support integrating oral health promotion into broader child nutrition and health strategies. Addressing oral health in childhood may therefore contribute not only to improved dental outcomes but also to better overall growth and development.

## Figures and Tables

**Table 1:** Characteristics of children aged 6–9 years (N = 120,994).

Characteristic	Value
Age (years), mean ± SD	7.52 ± 1.11
Male sex, n (%)	60,786 (50.2)
Urban residence, n (%)	76,831 (63.5)
Anthropometry	
WHZ, mean ± SD	−0.04 ± 1.00
BMI, mean ± SD	16.73 ± 2.30
HAZ, mean ± SD	0.01 ± 1.00
WAZ, mean ± SD	0.00 ± 1.00
Oral health	
Extracted teeth, median (IQR)	0 (0–1)
Caries index (dmft), median (IQR)	2 (0–5)

Values are mean ± SD or median (interquartile range) unless otherwise indicated.

**Table 2:** Anthropometric and oral health indicators by age among children aged 6–9 years (N = 120,994).

Indicator	Age 6 (n = 28,756)	Age 7 (n = 30,788)	Age 8 (n = 31,097)	Age 9 (n = 30,353)	*p*-Value
Anthropometry					
HAZ, mean ± SD	0.01 ± 1.00	0.02 ± 1.00	0.01 ± 1.00	0.02 ± 1.00	0.551
WAZ, mean ± SD	0.00 ± 1.00	0.00 ± 1.00	0.00 ± 1.00	0.00 ± 1.00	0.998
WHZ, mean ± SD	−0.11 ± 0.96	−0.04 ± 1.01	−0.01 ± 1.02	−0.01 ± 1.02	<0.001
BMI, mean ± SD (kg/m^2^)	16.26 ± 1.89	16.41 ± 2.03	16.89 ± 2.27	17.51 ± 2.59	<0.001
Oral health					
Decayed teeth, mean ± SD	2.60 ± 3.02	2.39 ± 2.69	2.13 ± 2.40	1.78 ± 2.14	<0.001
Filled teeth, mean ± SD	0.65 ± 1.38	0.69 ± 1.37	0.64 ± 1.27	0.56 ± 1.16	<0.001
Extracted teeth, mean ± SD	0.17 ± 0.65	0.22 ± 0.71	0.24 ± 0.72	0.24 ± 0.72	<0.001
DMFT index, mean ± SD	3.42 ± 3.46	3.29 ± 3.14	3.00 ± 2.85	2.58 ± 2.58	<0.001

Values are mean ± SD. *p*-values from one-way ANOVA.

**Table 3:** Mean anthropometric indicators by extracted teeth category.

Outcome	Extracted Teeth Category			*p*-Value
	0 Teeth (n = 105,882)	1–2 Teeth (n = 12,649)	≥3 Teeth (n = 2463)	
BMI	16.74 ± 2.30	16.78 ± 2.26	16.72 ± 2.17	0.507
WAZ	0.00 ± 1.00	−0.02 ± 0.99	−0.04 ± 0.94	0.004
HAZ	0.01 ± 1.00	0.01 ± 0.99	0.01 ± 0.98	0.687
WHZ	−0.03 ± 1.00	−0.06 ± 0.99	−0.09 ± 0.94	0.001

Values are mean ± SD. *p*-values from one-way ANOVA.

**Table 4:** Association between tooth loss category and anthropometric outcomes among children aged 6–9 years.

Outcome	Tooth Loss Category	Unadjusted β (95% CI)	*p*-Value	Adjusted β ^1^ (95% CI)	*p*-Value
WHZ	0 teeth	0 (Reference)	-	0 (Reference)	-
	1–2 teeth	−0.026 (−0.045, −0.008)	0.005	−0.025 (−0.047, −0.004)	0.019
	≥3 teeth	−0.052 (−0.091, −0.012)	0.013	−0.058 (−0.084, −0.032)	<0.001
WAZ	0 teeth	0 (Reference)	-	0 (Reference)	-
	1–2 teeth	−0.026 (−0.045, −0.008)	0.005	−0.026 (−0.045, −0.008)	0.005
	≥3 teeth	−0.040 (−0.080, −0.001)	0.049	−0.040 (−0.080, −0.001)	0.049

^1^ Adjusted for age (years) and sex. The tooth loss category was entered as a set of dummy variables in linear regression models, with children without extracted teeth as the reference group. β represents unstandardized regression coefficients.

## Data Availability

The data presented in this study are available on request from the corresponding author due to ethical approval restrictions.
